# Procedural characteristics and clinical outcomes from same-day discharge after pulsed field ablation treatment for atrial fibrillation: an admIRE trial sub-analysis

**DOI:** 10.1093/europace/euaf270

**Published:** 2025-11-15

**Authors:** Luigi Di Biase, Vivek Y Reddy, Xiaodong Zhang, David Newton, Sandeep Goyal, Devi Nair, William H Sauer, Vivek Iyer, Christopher F Liu, Jose Osorio, Moussa Mansour, Hugh Calkins, Oussama Wazni, Andrea Natale

**Affiliations:** Cardiac Arrhythmia Center, Division of Cardiology at the Montefiore Medical Center, Albert Einstein College of Medicine, 111 East 210 Street, New York, NY 10467, USA; Helmsley Electrophysiology Center, Mount Sinai Fuster Heart Hospital, NewYork, NY, USA; Cardiac Arrhythmia Center, Division of Cardiology at the Montefiore Medical Center, Albert Einstein College of Medicine, 111 East 210 Street, New York, NY 10467, USA; Memorial Health University Medical Center, Savannah, GA, USA; Piedmont Heart Institute, Atlanta, GA, USA; St. Bernards Medical Center & Arrhythmia Research Group, Jonesboro, AR, USA; Cardiac Arrhythmia Service, Brigham and Women’s Hospital and Harvard Medical School, Boston, MA, USA; MarinHealth Medical Center, Larkspur, CA, USA; New York Presbyterian-Weill Cornell Medicine, NewYork, NY, USA; HCA Florida Miami, FL, USA; Massachusetts General Hospital, Boston, MA, USA; Johns Hopkins Medical Institutions, Baltimore, MD, USA; Cleveland Clinic Foundation, Cleveland, OH, USA; Texas Cardiac Arrhythmia Research Foundation, Austin, TX, USA; Department of Biomedicine and Prevention, Division of Cardiology, University of Tor Vergata, Rome, Italy

**Keywords:** Atrial fibrillation, Catheter ablation, Pulsed field ablation, Pulmonary vein isolation, Same-day discharge

## Abstract

**Aims:**

Same-day discharge (SDD) following catheter ablation for atrial fibrillation (AF) yields a promising approach to enhance patient satisfaction and reduce healthcare costs. While prior data support the safety of SDD after radiofrequency ablation, evidence following pulsed field ablation (PFA) remains limited. This sub-analysis of the admIRE trial (NCT05293639) evaluates the safety of SDD compared with overnight stays (ONS) after PFA using a variable-loop circular catheter (VLCC).

**Methods and results:**

Baseline and procedural characteristics, safety, and effectiveness were compared between SDD and ONS groups. Primary effectiveness was defined as freedom from composite failure using Kaplan–Meier estimates. Serious adverse events (SAEs) occurring ≤7 days and ≥1 day post-index procedure were assessed. Amongst 277 patients in the admIRE trial, 119 (43.0%) completed SDD, and 158 (57.0%) stayed overnight. The SDD group included fewer patients aged ≥65 years (41.2% vs. 52.5%), shorter fluoroscopy times (4.0 vs. 8.9 min), higher first-pass isolation rates (97.5% vs. 82.9%), and fewer procedural AEs (1.7% vs. 3.8%). More procedures in the SDD group occurred in the morning (63.0% vs. 39.2%). Freedom from primary effectiveness failure was similar (SDD, 75.4% [95% CI 67.4–83.3%] vs. ONS, 74.0% [95% CI 66.4–81.5%]). No significant difference in SAE risk was observed overall [HR, 1.07 (95% CI 0.47–2.44)] or ≤7 days post-procedure [HR, 0.78 (95% CI 0.17–3.49)]. Post-procedural serious cardiac/vascular AE rates were also comparable (SDD, 2.5% vs. ONS, 1.3%).

**Conclusion:**

SDD is feasible in a paroxysmal AF population undergoing PFA with the VLCC, demonstrating comparable safety and effectiveness outcomes with overnight hospitalization.

## Graphical Abstract

**Graphical Abstract:**
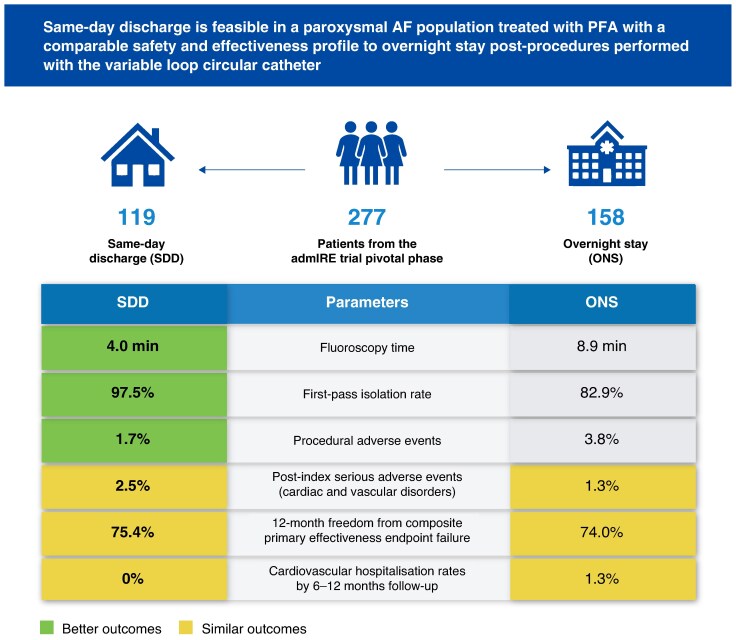
AF, atrial fibrillation; PFA, pulsed field ablation. The figure is courtesy of Biosense Webster, Inc., part of Johnson & Johnson MedTech. All rights reserved.

What’s new?

- This *ad hoc* sub-analysis of patients from the admIRE trial of pulsed field ablation (PFA) for atrial fibrillation (AF) compared patients who completed same-day discharge (SDD) and those admitted for an overnight stay (ONS); rates of 12-month freedom from composite primary effectiveness failure were similar for the SDD group and ONS group.
- No statistically significant association for serious adverse event risk was observed by discharge status.
- This was the first prospective, United States Food and Drug Administration (FDA) investigational device exemption clinical trial to study SDD in a cohort of patients undergoing PFA for AF; the comparable safety and effectiveness outcomes demonstrate the feasibility of SDD discharge with PFA.

## Introduction

Pulmonary vein isolation serves as the standard catheter-based treatment for atrial fibrillation (AF) and is performed using various energy modalities, including radiofrequency and cryoballoon ablation. Recently, pulsed field ablation (PFA) has emerged as a novel technique that offers improved tissue selectivity and a promising safety and efficacy profile that is comparable with earlier ablation modalities.^1,2^ In this context, an innovative integrated PFA system, incorporating non-fluoroscopic guidance through online electroanatomical mapping and tissue-to-catheter proximity feedback, was developed to optimise procedural safety and efficacy.

Initial evaluation of this integrated PFA system occurred through the inspIRE trial (ClinicalTrials.gov Identifier: NCT04524364), which assessed 12-month post-ablation clinical outcomes in European and Canadian patients.^3^ This study was followed by the US admIRE trial (ClinicalTrials.gov Identifier: NCT05293639),^4^ which enrolled 277 patients with paroxysmal AF (PAF) across 30 centres, and confirmed this system’s safety and effectiveness through demonstrated high acute efficacy (short procedure duration), low complication rates, and reduced fluoroscopy exposure.^4^

Same-day discharge (SDD) following AF ablation has gained interest as a strategy to improve healthcare efficiency, reduce hospital costs and patient ablation wait times, and enhance overall patient satisfaction.^5–11^ It has been increasingly utilized in the electrophysiology community long before the advent of PFA.^12–14^ Historically, patients undergoing catheter ablation for AF have been observed overnight due to the procedural complexity and the perceived risk of post-procedural complications. However, increasing evidence supports that SDD is both safe and feasible in patients who undergo an evaluation of key factors, including: (1) stable anticoagulation, (2) absence of bleeding history, (3) no systolic heart failure, (4) no history of pulmonary disease, (5) no recent cardiac interventions within 60 days of ablation, (6) body mass index (BMI) < 35 kg/m^2^, and (7) acceptable CHA_2_DS_2_-VASc stroke risk (typically ≤3).^15^ Additionally, SDD is more likely to be implemented for procedures performed earlier in the day and under conscious sedation.

Despite these considerations, there are limited data evaluating SDD in the context of PFA, particularly with newer integrated systems. Existing studies examining SDD in catheter ablation are often observational, which can introduce biases related to patient and site selection.^15^ For example, some studies indicate that procedures for participants assigned to SDD were shorter, were more likely to be repeat ablations, were generally performed under sedation rather than general anaesthesia, and were less likely to involve linear lesions or electrical cardioversion.^16^ Consequently, the results may show an apparent advantage in procedural success and the safety profile for SDD, which can complicate interpretation. Conversely, long-term safety and efficacy of SDD is comparable with that of overnight stays (ONS) regardless of the ablation modality employed, whether radiofrequency or balloon-based techniques.^17^

This *ad hoc* analysis of the admIRE trial was conducted to evaluate the safety and effectiveness of SDD compared with ONS following PFA procedures using the variable-loop circular catheter (VLCC). By assessing real-world procedural and clinical outcomes, this analysis aims to inform best practices for post-procedural care in the era of next-generation ablation technologies.

## Methods

### Study design and ablation procedure

The admIRE trial (ClinicalTrials.gov Identifier: NCT05293639) was a multicentre, prospective, non-randomized, single-arm interventional study that assessed the safety and effectiveness of a variable-loop PFA catheter (VARIPULSE Catheter, Biosense Webster, Inc., part of Johnson & Johnson MedTech, Irvine, CA, USA) with an integrated electroanatomical mapping (EAM) system (CARTO 3 System, Biosense Webster, Inc., part of Johnson & Johnson MedTech). Full details of the admIRE trial study design and ablation procedure have been previously reported.^4^ The institutional ethics review boards at each participating study centre approved the study, and the enrolled participants provided informed consent prior to undergoing any study procedures.

### Patient follow-up and monitoring

Monitoring for arrhythmia recurrence was previously described in detail.^4^ Transtelephonic monitoring (KardiaMobile 6L) was performed weekly from Months 1 to 5, monthly from Months 6 to 12, and for symptomatic events.

### Statistical analysis

Freedom from recurrence at 12 months was estimated using the Kaplan–Meier method. A Cox proportional-hazards regression model, with incidence of SDD as the primary factor and adjusted for age, sex, and BMI, was used to estimate hazard ratio (HR) and 95% confidence interval (CI) for serious adverse events (SAEs) occurring 1–7 days and ≥1 day post-index procedure. Demographic results are presented as mean (±SD), and procedural results are presented as median (quartile 1–quartile 3). A mixed-effect logistic regression analysis, treating the study centre as a random effect, was performed to assess the association of various factors with SDD. Univariable logistic regressions were first performed to assess the association between variables of interest and SDD. The *P*-value obtained from the Wald χ^2^ statistic for each factor served as a screening criterion to identify variables for inclusion in the multivariable logistic regression model. A *P*-value < 0.20 was used as the cutoff for screening covariates. A final multivariable logistic regression model was constructed by incorporating all variables that met the screening criterion of *P*-value < 0.20 in the univariable analysis and retained a *P*-value < 0.20 when modelled jointly. If strong multicollinearity was observed, variables were prioritized based on relevance and interpretability in the model.

Changes in cardiovascular (CV) hospitalization and direct current cardioversion (DCCV) at 6–12 months post-index procedure vs. baseline were assessed via exact McNemar’s test. A continuity correction was applied where needed. Statistical analyses were performed using SAS 9.4 or SAS Studio 3.8 (SAS Institute, Inc.).

## Results

**Figure 1:**
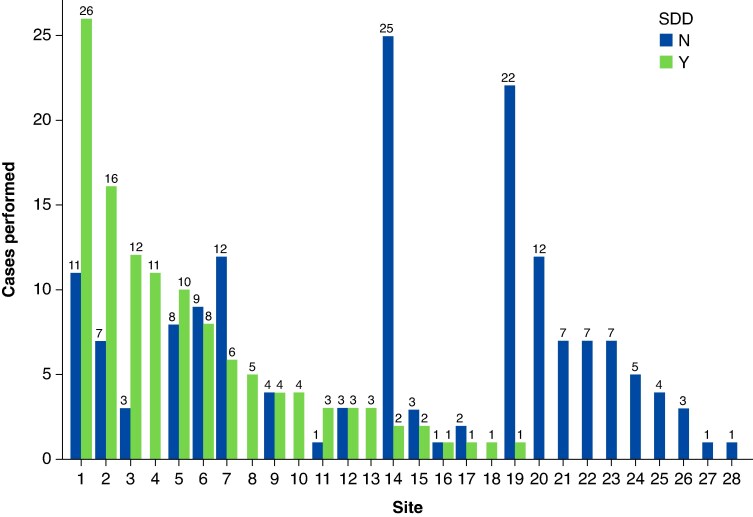
Same-day discharge vs. ONS patient enrolment by site. ONS, overnight stay; SDD, same-day discharge. The figure is courtesy of Biosense Webster, Inc., part of Johnson & Johnson MedTech. All rights reserved.

The retrospective sub-analysis included 277 patients from the pivotal phase of the admIRE trial. Of these, 119 (43.0%) patients completed SDD, with 113 (95.0%) patients originally planned for SDD. In comparison, 158 (57.0%) patients were admitted for ONS, with 126 (79.7%) patients initially planned for ONS. Site-level distributions of SDD and ONS are described in *Figure 1*.

Amongst the 119 participants in the SDD cohort, 78 (65.5%) patients were male and 41 (34.5%) were female. Meanwhile, the ONS cohort (*n* = 158) was composed of 100 male (63.3%) and 58 female (36.7%) patients. Median (interquartile range) BMI was 28.1 (24.9–32.1) kg/m^2^ in the SDD group and 27.8 (24.5–31.7) kg/m^2^ in the ONS group. Fewer patients in the SDD group were aged ≥ 65 years compared with the ONS group (41.2% vs. 52.5%). Demographic data comparing the SDD and ONS groups are presented in *Table 1*.

**Table 1:** Baseline characteristics

Characteristics^a^	SDD (*n* = 119)	ONS (*n* = 158)	*P*-value
Age, years	62.0 (55.0–68.0)	65.0 (59.0–69.0)	0.03
Female	41 (34.5)	58 (36.7)	0.71
Body mass index, kg/m^2^	28.1 (24.9–32.1)	27.8 (24.5–31.7)	0.63
LVEF, %	60.0 (55.0–65.0)	60.0 (60.0–65.0)	0.03
Left atrial diameter, mm	38.0 (34.0–42.0)	39.0 (34.0–43.0)	0.83
CHA_2_DS_2_-VASc	2.0 (1.0–2.0)	2.0 (1.0–3.0)	0.70
PAF history, mo	24.0 (9.0–72.0)	24.0 (11.0–60.0)	0.60
Myocardial infarction	5 (4.2)	3 (1.9)	0.30
Hypertension	67 (56.3)	81 (51.3)	0.47
Type 2 diabetes	15 (12.6)	15 (9.5)	0.44
Coronary disease	30 (25.2)	25 (15.8)	0.07
Obstructive sleep apnoea	33 (27.7)	44 (27.8)	>0.99
Thrombo-embolic events	4 (3.4)	6 (3.8)	>0.99
Congestive heart failure	5 (4.2)	4 (2.5)	0.50
NYHA Class I	2 (1.7)	1 (0.6)	>0.99
NYHA Class II	3 (2.5)	3 (1.9)	
Number of failed AADs	1.0 (1.0–1.0)	1.0 (1.0–2.0)	0.19
Class I/III AAD	1.0 (1.0–1.0)	1.0 (1.0–1.0)	0.80

AAD, antiarrhythmic drug; CHA_2_DS_2_-VASc, congestive heart failure, hypertension, age ≥ 75 years (doubled), type 2 diabetes, previous stroke or transient ischaemic attack (doubled), vascular disease, age 65–74 years, and sex category; IQR, interquartile range; LVEF, left ventricular ejection fraction; NYHA, New York Heart Association; ONS, overnight stay; PAF, paroxysmal atrial fibrillation; SDD, same-day discharge.

^a^Data are presented as median (IQR) or *n* (%).

A single transseptal access was utilized for 100% of the SDD cases, while 86.1% of the ONS cases underwent single transseptal access, and 13.9% used double transseptal access. Median (interquartile range) total mapping time was 6 (4.0–10.0) min for the SDD group and 7 (4.0–10.0) min for the ONS group. Median total duration of catheter dwell time in the left atrium was 39.0 (30.0–53.0) and 40 (33.0–53.0) min for the SDD and ONS groups, respectively. The number of catheter exchanges during the procedure was also similar between groups, with 2 (2–2) exchanges for the SDD group and 2 (2–3) exchanges for the ONS group. Median fluoroscopy time was significantly shorter in the SDD group [4.0 (0.0–8.7) min] compared with the ONS group [8.9 (2.5–19.1) min]. Additionally, the first-pass isolation rate was higher in the SDD group vs. the ONS group (97.5% vs. 82.9%), and procedural adverse events (AEs) were less frequent (1.7% vs. 3.8%). A greater proportion of SDD procedures were performed in the morning compared with ONS (63.0% vs. 39.2%). Procedural data comparing the SDD and ONS groups are provided in *Table 2*. A transthoracic echocardiogram was performed prior to discharge to evaluate pericardial effusion, and all participants had direct access to the treating physician in case of complications after discharge.

**Table 2:** Procedural characteristics

Characteristics^a^	SDD (*n* = 119)	ONS (*n* = 158)	*P*-value
General anaesthesia used	118 (99.2)	158 (100.0)	0.43
Single transseptal access	119 (100.0)	136 (86.1)	<0.001
Procedure time, min	92.0 (67.0–114.0)	86.5 (64.0–123.0)	0.84
Total mapping time, min	6.0 (4.0–10.0)	7.0 (4.0–10.0)	0.90
PFA application time,^b^ s	16.2 (13.8–20.1)	17.5 (15.3–21.8)	<0.001
Fluoroscopy time, min	4.0 (0.0–8.7)	8.9 (2.5–19.1)	<0.001
Cases in which ICE was used	116 (97.5)	145 (91.8)	0.07
Cases with only the study catheter for mapping	11 (9.2)	24 (15.2)	0.15
Number of valid PFA applications per patient	67.0 (57.0–83.0)	72.0 (63.0–90.0)	<0.001
Received additional posterior wall segmental ablation	5 (4.2)	11 (7.0)	0.44

ICE, intracardiac echocardiography; IQR, interquartile range; ONS, overnight stay; PFA, pulsed field ablation; SDD, same-day discharge.

^a^Data are presented as median (IQR) or *n* (%); *n* = 158 for anaesthesia and *n* = 157 for all other parameters in the ONS group.

^b^Excludes idle time when PFA is not applied.

**Figure 2:**
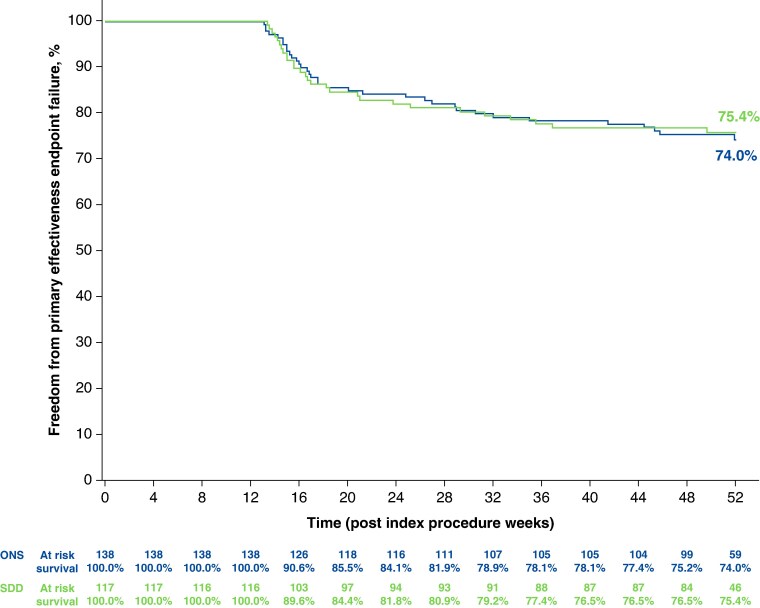
12-month freedom from primary effectiveness endpoint failure in the SDD vs. ONS groups. ONS, overnight stay; SDD, same-day discharge. The figure is courtesy of Biosense Webster, Inc., part of Johnson & Johnson MedTech. All rights reserved.

Twelve-month freedom from composite primary effectiveness failure was similar between groups: 75.4% (67.4–83.3%) in the SDD group and 74.0% (66.4–81.5%) in the ONS group (*Figure 2*). No statistically significant association for SAE risk (any safety event leading to death, serious deterioration in the patient’s health, or foetal distress, if applicable) was observed by discharge status (SDD or ONS), either overall [HR, 1.07 (95% CI 0.47–2.44)] or within 7 days post-procedure [HR, 0.78 (95% CI 0.17–3.49)]. Post-index SAEs for cardiac and vascular disorders were also comparable between groups: 2.4% for SDD vs. 1.3% for ONS; all were deemed not related to study devices or procedure.

Amongst the 32 planned SDD cases that did not result in SDD, the most common reasons were procedural AEs (31.3%) and patient preference (25.0%). Amongst patients who were discharged the same day, none experienced primary AEs. In contrast, the ONS group reported 9 events across 8 patients, including cardiac tamponade (1.9%), major vascular access complication/bleeding (1.3%), stroke/cerebrovascular accident (1.3%), pericarditis (0.6%), and transient ischaemic attack (0.6%; *Table 3*).

**Table 3:** Primary adverse events in the SDD vs. ONS groups

Primary adverse event^a^	SDD (*n* = 119)	ONS (*n* = 158)
Device- or procedure-related death	0 (0)	0 (0)
Atrio-oesophageal fistula	0 (0)	0 (0)
Cardiac tamponade or perforation	0 (0)	3 (1.9)
Major vascular access complication/bleeding	0 (0)	2 (1.3)
Myocardial infarction	0 (0)	0 (0)
Pericarditis	0 (0)	1 (0.6)
Heart block	0 (0)	0 (0)
Phrenic nerve paralysis (permanent)	0 (0)	0 (0)
Pulmonary vein stenosis	0 (0)	0 (0)
Stroke/cerebrovascular accident	0 (0)	2 (1.3)
Thromboembolism	0 (0)	0 (0)
Transient ischaemic attack	0 (0)	1 (0.6)

ONS, overnight stay; SDD, same-day discharge.

^a^Data are presented as *n* (%).

**Figure 3:**
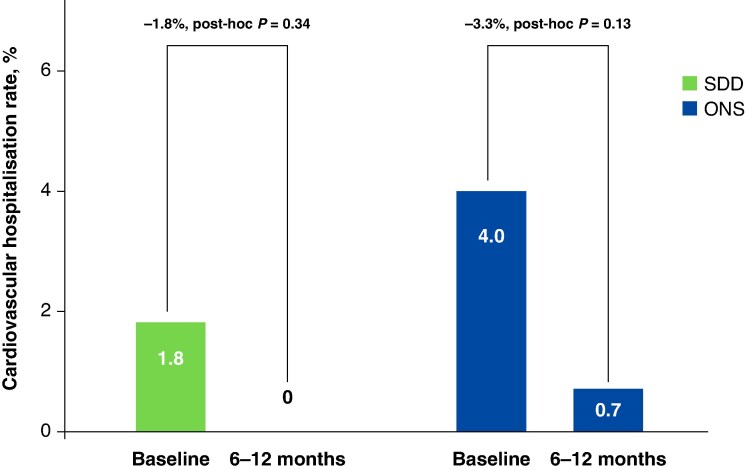
Cardiovascular hospitalization rates (*n* = 277). Visualization of percentage of CV hospitalization at baseline (up to 6 months prior to enrolment) through the 6- to 12-month follow-up period. CV, cardiovascular; ONS, overnight stay; SDD, same-day discharge. Baseline is defined as events happening within 6 months prior to enrolment. The figure is courtesy of Biosense Webster, Inc., part of Johnson & Johnson MedTech. All rights reserved.

**Figure 4:**
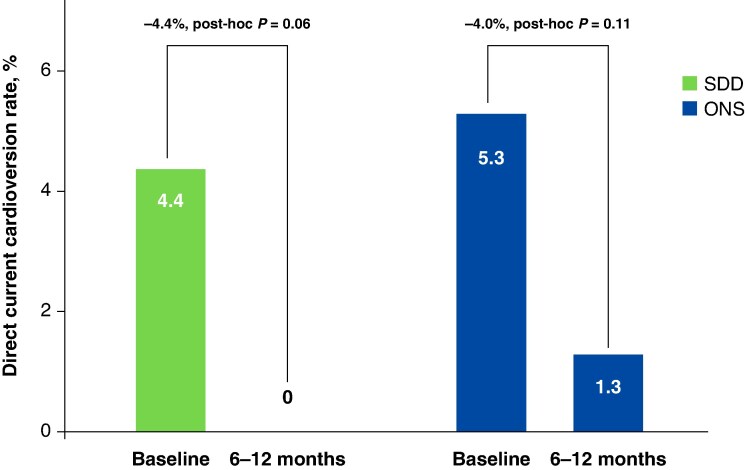
Direct current cardioversion rates (*n* = 277). Cardioversion use at baseline (up to 6 months prior to enrolment) compared with the 6- to 12-month follow-up period. ONS, overnight stay; SDD, same-day discharge. Baseline is defined as events happening within 6 months prior to enrolment. The figure is courtesy of Biosense Webster, Inc., part of Johnson & Johnson MedTech. All rights reserved.

In terms of healthcare utilization, the SDD group had lower CV hospitalization rates: 1.8% at baseline (up to 6 months prior to enrolment), decreasing to 0% by 6–12 months. Comparatively, the ONS group had a baseline hospitalization rate of 4.0%, which also declined over the same time period (*Figure 3*). Furthermore, DCCV was more common in the SDD cohort at baseline up to 6 months prior to enrolment (4.4%) and 0% at 6–12 months. In contrast, the ONS group recorded 5.3% at baseline and 1.3% at 6–12 months (*Figure 4*). Notably, only 1 hospital readmission (0.9%) occurred in the SDD group within 30 days post-index ablation procedure, whereas no readmissions (0%) were reported in the ONS group during that same time frame.

Repeat ablation was performed in 12 of 119 (10.1%) patients in the SDD group, and 13 of 157 (8.3%) patients in the ONS group. Most of these repeat ablations occurred within the evaluation period (Days 91–365: SDD, 83.3%; ONS, 100%). Of the 13 repeat ablations in the ONS group, 4 (30.8%) constituted second ablations, whereas no patients in the SDD group underwent a second ablation.

In a multivariable logistic regression analysis, patients in the SDD cohort were significantly less likely to be aged ≥65 years [OR, 0.47 (95% CI 0.22-0.98); *P* = 0.05], to have symptomatic AF episodes lasting ≥ 1 h [OR, 0.44 (95% CI 0.20-0.98); *P* = 0.04], and to present with high systolic blood pressure > 120 mm Hg [OR, 0.40 (95% CI 0.17-0.94); *P* = 0.04]. In addition, the SDD group was associated with shorter total ablation durations and more frequent morning procedures compared with the ONS group (*Table 4*). Additionally, the SDD cohort tended toward fewer procedural SAEs [OR, 0.10 (95% CI 0.01-1.13); *P* = 0.06].

**Table 4:** Logistic regression analysis for SDD vs. ONS

Variable	Univariable analysis		Multivariable analysis	
	OR (95% CI)	*P*-value	OR (95% CI)	*P*-value
Age (≥65 vs. <65 years)	0.63 (0.34, 1.15)	0.13	0.47 (0.22, 0.98)	0.05
Length of symptomatic AF episode (≥ 1 vs. <1 h)	0.57 (0.29, 1.11)	0.10	0.44 (0.20, 0.98)	0.04
Systolic blood pressure (>120 vs. ≤120 mm Hg)	0.48 (0.23, 1.00)	0.05	0.40 (0.17, 0.94)	0.04
Total ablation duration (>25–31 vs. ≤25 min)	0.30 (0.12, 0.79)	0.01	0.28 (0.09, 0.84)	0.03
Total ablation duration (>31–41 vs. ≤25 min)	0.43 (0.16, 1.12)	0.01	0.33 (0.11, 1.00)	0.03
Total ablation duration (>41 vs. ≤25 min)	0.18 (0.06, 0.51)	0.01	0.17 (0.05, 0.58)	0.03
Procedural SAE (yes vs. no)	0.17 (0.02, 1.12)	0.07	0.10 (0.01, 1.13)	0.06
Morning procedure (yes vs. no)	5.88 (2.89, 11.99)	0.000	8.92 (3.81, 20.88)	<0.001

AF, atrial fibrillation; CI, confidence interval; ONS, overnight stay; OR, odds ratio; SAE, serious adverse event; SDD, same-day discharge.

## Discussion

This sub-analysis of the admIRE pivotal trial presents several key findings: (1) it is the first prospective, United States Food and Drug Administration (FDA) investigational device exemption clinical trial to demonstrate the feasibility of SDD in a large cohort of patients undergoing PFA for AF; (2) patients in the SDD group were generally younger, had more procedures scheduled in the morning, and underwent single transseptal access with similar mapping time and catheter dwell time in the left atrium, but shorter fluoroscopy duration in comparison with ONS; and (3) SDD, after initial experiences with PFA for AF, was shown to be safe, with no significant difference in primary safety outcomes compared with ONS.

With the advancement of AF ablation tools and techniques, overall complication rates have decreased precipitously.^18^ In the recent decade, there has been increasing interest in expanding SDD protocols beyond diagnostic coronary angiography or device implantation to include AF ablation. However, barriers to SDD in AF include the procedural complexity, the need for general anaesthesia or deep sedation, and requirements for femoral vascular access monitoring. Previous reports have identified non-clinical logistical factors (43%), prolonged post-procedure recovery (42%), and minor procedural complications (15%) as the prominent obstacles to SDD.^18^ Nonetheless, multiple observational studies have demonstrated the safety and feasibility of SDD in low-risk patients without intraprocedural or immediate post-procedural complications.^16,17,19–23^ A meta-analysis of 12 studies involving > 18 000 ablations found no significant increase in major complication rates with SDD compared with ONS.^6^ Additionally, US propensity score–matched data from a readmissions database revealed a similar 30-day readmission rate and reduced healthcare costs in the SDD group.^24^ Moreover, two large single-arm cohort studies observed that SDD cases were more likely to be performed using conscious sedation over general anaesthesia, with a majority of patients reporting high satisfaction.^16,25^

Our prospective study with predefined, stringent-monitoring protocols and close data oversight provides a higher level of data integrity, which represents a clear advantage and sets the study apart from prior reports that relied on voluntary self-reporting. We found no statistically significant difference in SAE rates between SDD and ONS, both overall and within 7 days post-ablation, confirming the safety profile of the PFA system. Importantly, a multivariate logistic regression analysis showed that SDD patients were less likely to experience same-day SAEs, further reinforcing the safety and practicality of SDD following PFA.

While existing literature reports similar safety profiles in patients able to achieve SDD compared with ONS, it is important to note that complications are major obstacles preventing planned SDD. In our study, amongst 32 patients planned for SDD, the main reasons for not achieving SDD included experiencing AEs in 31.3% of cases. Most previous experiences using thermal energy ablations were not able to achieve 100% planned SDD, almost always due to complications.^5–11^ Fortunately, several studies demonstrated that most serious complications, and those requiring hospital admission, occurred either during the procedure or within 6 h post-ablation. There were very few complications that occurred between 6 h post-ablation and prior to discharge in patients with ONS.^26,27^ Patients without complications after observation who opted for ONS had no difference in 30-day readmission compared with those who were discharged the same day.^26,27^ Conversely, patients who experienced procedural complications and stayed overnight had almost twice the risk of readmission.^7^ This further validates that only patients with complications observed during the 4–6 h post-op could truly benefit from overnight hospital monitoring.

We believe that SDD can potentially be the default pre-procedural plan in low-risk patients. The Heart Rhythm Society and the American College of Cardiology have developed guidelines for SDD to improve patient outcomes during standard-of-care practice.^28^ Of note, these cases should ideally be planned in the morning to allow for vascular monitoring post-procedure. The absence of intraprocedural complications during AF ablation is indeed a strong predictor of safety with SDD, given the above findings. Patients without intraoperative or immediate post-op complications should undergo SDD. Overnight hospital stays are ideally reserved for patients who have suffered from post-op complications. Nevertheless, we recognise that the feasibility of implementing SDD outside of the United States is not purely determined by clinical safety and efficacy. Structural and economic factors, particularly reimbursement policies, differ considerably between healthcare systems and can substantially influence practice. For example, in several European countries, reimbursement is reduced considerably if patients are discharged on the same day, which can act as a practical barrier to adopting SDD despite clear evidence of safety.^9^

Identifying the right patient population for SDD is key for successful implementation. Our multivariate logistic regression analysis demonstrated that patients in the SDD group were more likely to be aged <65 years, had <1 h of symptomatic AF episode length, and a systolic blood pressure ≤ 120 mm Hg compared with the ONS cohort. In addition, patients in the SDD group were significantly more likely to have morning procedures and shorter total ablation durations compared with their ONS counterparts.

Several centres shared their experience on the eligibility criteria and workflow for achieving effective SDD. The most commonly cited criteria include: stable use of anticoagulation, no bleeding history, non-severely reduced left ventricular systolic function (usually left ventricular ejection fraction > 35–40%), no pulmonary disease, BMI < 35 kg/m^2^, close proximity to hospital, and adequate home support.^15,29^ Protocolized SDD workflows, such as those from the REAL AF registry and Spanish centres utilizing a dedicated SDD co-ordinator, have demonstrated success in achieving >85–90% SDD rates in eligible candidates.^29,30^

Sometimes, patients prefer ONS for better monitoring due to a lack of social support at home after SDD. In our study, a quarter of patients did not achieve planned SDD due to personal preference. A German survey found that 50% of patients were willing to incorporate SDD into their care, with the rest of the patients expressing concerns about how to manage post-op symptoms, and inadequate recognition and treatment of complications from home.^31^ Another European survey observed higher SDD implementation in tertiary and higher volume centres (83.8% and 85.3%, *P* < 0.01) while maintaining low complication and rehospitalization rates.^32^ In addition, the advent of PFA is increasingly allowing the incorporation of SDD as electrophysiologists become more comfortable with the latest technologies. For example, the MANIFEST Registry reported 15.1% of patients being discharged same day.^33^ This study reports the early applications using this specific PFA technology. Further experiences may potentially produce higher SDD rates. It is our job as attending physicians to counsel and reassure eligible patients about the safety of SDD to promote more efficient AF ablation workflows, particularly in the PFA era.

### Limitations

This retrospective *ad hoc* analysis focuses on a subgroup of a larger, prospective, protocol-defined, single-arm study that was not powered to study the benefits of SDD. To facilitate a more robust interpretation and a clearer understanding of the differences between SDD and ONS patients, a randomized controlled trial with larger sample sizes is necessary. The reported *P*-values should be interpreted with caution and considered hypothesis-generating rather than confirmatory.

Furthermore, the choice between SDD and ONS cases was not predefined in the study protocol and depended on the preferences of the operator and/or the institution. There were no consistent patterns in the types of complications or other characteristics of the cases, so decisions were often guided by the standard procedures of the institution.

Additionally, evaluation of patient perspectives was beyond the scope of the present sub-analysis. Future studies should ideally incorporate patient-reported outcomes and perspectives, as recent European surveys have shown that, while many patients are receptive to SDD, concerns remain regarding recognition and management of complications at home, as well as variability in practice across healthcare systems.^31^

## Conclusion

SDD is feasible in a PAF population treated with VLCC PFA with a comparable safety and effectiveness profile with ONS procedures.

## Data Availability

Johnson & Johnson MedTech has an agreement with the Yale Open Data Access (YODA) Project to serve as the independent review panel for the evaluation of requests for clinical study reports and patient-level data from investigators and physicians for scientific research that will advance medical knowledge and public health. Requests for access to the study data can be submitted through the YODA Project site at http://yoda.yale.edu.
